# Hearing Loss and Social Isolation in Community-Dwelling Older Adults: The Role of Neighborhood Disorder and Perceived Social Cohesion

**DOI:** 10.3390/ijerph22040583

**Published:** 2025-04-08

**Authors:** Sol Baik, Kyeongmo Kim

**Affiliations:** 1 Weldon Cooper Center for Public Service, University of Virginia, Charlottesville, VA 22903, USA; 2 School of Social Work, Virginia Commonwealth University, Richmond, VA 23284, USA; kkim7@vcu.edu

**Keywords:** hearing loss, social cohesion, social isolation, neighborhood disorder

## Abstract

Hearing loss is one of the most common sensory impairments acquired with aging. This condition causes communication difficulties, leading to social isolation, dependence on others, and a reduced quality of life. However, less is known about the influence of environmental factors on the experiences of older adults with hearing loss. This study utilized three waves of the National Health and Aging Trends Study (2011–2013), analyzing data from 3950 community-dwelling older adults. Survey-weighted random intercept models were used to investigate whether hearing loss is associated with social isolation over the three waves and whether this relationship is moderated by neighborhood disorder and perceived neighborhood social cohesion. The study found that older adults with hearing loss were significantly less socially isolated, while the perceived social cohesion significantly moderated the effect of hearing loss on social isolation. Given that hearing function deteriorates with age and hearing aids or other devices are rarely covered by third-party payers, except for some state Medicaid plans or rehabilitation services for veterans, addressing modifiable neighborhood factors may be the most effective way to help individuals remain socially engaged and avoid isolation.

## 1. Introduction

Hearing loss is one of the most common sensory impairments that people acquire as they age [1,2,3]. Age is the strongest predictor for hearing loss among adults aged 20 to 69, indicating that people tend to experience disturbance of their hearing function as they get older [4]. A recent report on the profile of older Americans indicated that approximately 14% of older adults experience serious hearing difficulties [5]. The rates of hearing loss increase with age: 2 percent of middle-aged adults aged 45 to 54, 8.5 percent of those aged 55 to 64, 25 percent of those between 65 and 74, and 50 percent of those above 75 have disabling hearing loss [6]. Hearing ability is one of the important personal resources in communicating with others and maintaining social relationships. Thus, hearing loss causes difficulties with communication, leading to social isolation, dependence on others, and a reduction in quality of life [7,8,9].

According to the Surgeon General’s report [10], social isolation and loneliness have been identified as urgent public health concerns in the U.S., posing serious risks to health and longevity. The report highlights that older adults and individuals with disabilities are particularly vulnerable to social isolation, with older adults with hearing loss facing an increased risk of experiencing social isolation. Despite the increasing concern about the social isolation of older adults and those with disabilities, the attention to interventions for hearing loss has mostly centered on improving hearing ability, rather than intervening in the social experiences or the environments surrounding the individuals. To mitigate the negative effect of hearing loss on social experiences, a few intervention studies found that traditional hearing aids or cochlear implantation improved social isolation and loneliness in older adults [11]. However, these interventions had limitations due to small sample sizes and the presence of confounding factors. Regarding observational studies using larger population-based datasets, most of the literature was restricted to cross-sectional studies [12]. Huang et al. [13] recently found a longitudinal influence of hearing impairment on greater odds of social isolation in older adults, indicating the limitation of being unable to capture the characteristics of the environments in which participants live.

A recent scoping review suggests that neighborhood places promote social engagement [14]. Neighborhood disorder can prevent older adults from participating in social activities, while a high level of neighborhood cohesion encourages it [15]. This is particularly relevant for older adults with hearing loss. Older adults with hearing loss in noisy environments are discouraged from social participation, whereas supportive environments where they feel understood foster engagement [16]. Still, few research studies have been conducted about the influence of environmental factors or the interaction between hearing loss and the environment on social isolation.

According to the ecological model of aging [17], individual variations in health and well-being result from the interplay between a person’s internal capabilities and their external environment. Environment broadly consists of five classes: physical, personal, small group, suprapersonal, and social/megasocial environment [17,18]. Among these components, the ecological model of aging empirically illustrates the physical environment, such as the neighborhood characteristics and housing environment, and the social environment in terms of social connections with close family and friends or in a neighborhood context [17,19]. Based on this theoretical model, people with limited personal resources (e.g., functional capacity) can age well if they have supportive environment-based resources to compensate for their limitations [20]. As such, the negative impact of hearing loss on the social functions of older adults can be mitigated through a physically and socially supportive environment, even though they may be socially isolated due to their increasing difficulty with communication.

Guided by the limitations and gaps in previous study findings and the theoretical framework of the ecological model of aging, this study addressed the following research questions: (1) Is there an association between hearing loss and social isolation among community-dwelling older adults over time? (2) Is the relationship between hearing loss and the social isolation of community-dwelling older adults moderated by neighborhood disorder and perceived social cohesion over time? This study hypothesized, regarding the first research question, that older adults with hearing loss experience higher levels of social isolation over three years compared to those without hearing loss. The second research hypothesis was that the negative impact of hearing loss on social isolation is moderated by neighborhood conditions, such that older adults living in less disordered and more cohesive communities experience lower levels of social isolation.

## 2. Materials and Methods

### 2.1. Data and Sample

This study used three waves of the National Health and Aging Trends Study (NHATS): Round 1 (*N* = 8245), Round 2 (*N* = 7075), and Round 3 (*N* = 5799), collected between 2011 and 2013. NHATS is a longitudinal study on late-life functioning with a nationally representative sample of Medicare beneficiaries aged 65 and older. The survey was conducted annually with the same cohort, data were publicly available, and datasets were deidentified by registering online (http://www.nhats.org, accessed on 1 July 2021). Through the application process, NHATS also provides sensitive data including demographic information, such as race and ethnicity. This current study combined three waves of publicly available datasets and the first wave of sensitive data for analyses. All sections of the questionnaires, except for the Facility Questionnaire and Interviewer Remarks, were administered to the sample persons or designated proxies [21]. The NHATS employed a stratified three-stage sampling design, selecting primary sampling units (counties), secondary sampling units (ZIP codes), and Medicare beneficiaries aged 65+, with oversampling of the oldest adults and non-Hispanic Black individuals for better representation [22]. The overall weighted response rates were 71.3% (Round 1), 85.3% (Round 2), and 87.4% (Round 3), respectively.

This study only included older adults aged 65 and above living in the community in all three rounds. Residents who lived in or transitioned to nursing homes or assisted living facilities were not included (*n* = 4478). Individuals who used proxy respondents in any of the three rounds were also excluded (*n* = 4121). The reasons for using the proxy were whether a sample person had dementia or a cognitive impairment, was too ill, had a speech impairment, had a hearing impairment, or was in other non-specified situations. After removing the cases with missing data in the analyses, this study had 3950 community-dwelling older adults as the analytic sample.

### 2.2. Measures

#### 2.2.1. Social Isolation

Social isolation, the dependent variable, was operationalized with the measure developed by Pohl et al. [23]. The measure was based on the Social Network Index (SNI) by Berkman [24] and Berkman and Syme [25]. They used NHATS items that could be comparable to SNI’s indicators of isolation: marriage or partnership; family and friends; church participation; and club participation. Four domains were measured, with six items. One point was recorded for a negative response on each item. The summation of all the items measured social isolation with a higher score, indicating greater isolation (range = 0 to 6). First, the domain of marriage and partnership was assessed using the following item: ‘Are you currently married, living with a partner, separated, divorced, widowed, or never married?’ Those who answered married or living with a partner were coded as 0 and others who responded as separated, divorced, widowed, or never married were entered as 1 (1 = No marriage or partner; 0 = Married or living with a partner). Second, the domain of contact with family and friends was divided into two aspects: having a conversation about important things (i.e., ‘Looking back over the past year, who are the people you talked with most often about important things?’) and visiting friends and family in person (i.e., ‘In the last month, did you ever visit in-person with friends or family not living with you either at home or theirs?’). The response to having an important talk was divided into two separate variables: family and friends. Those who had not talked to their family members over the past year were recorded as 1 and the same rule was applied to those who had not had an important conversation with friends (1 = No family/friends identified; 0 = Family/friends identified). The third domain regarding religious participation was also measured as a binary variable (1 = No, 0 = Yes) using the response to the following question: ‘In the past month, did you ever attend religious services?’ Lastly, the domain on club participation was recorded with the question, ‘In the past month, did you ever participate in clubs, classes, or other organized activities?’ (1 = No, 0 = Yes). The measure was validated by establishing convergent validity with depression risk and divergent validity with well-being [23].

#### 2.2.2. Hearing Loss

This study measured hearing loss, the independent variable, according to whether the participants reported any of the following: (1) using a hearing aid or other hearing device use in the last month, (2) inability to hear well enough to use the telephone, (3) inability to hear well enough to carry on a conversation in a room with a radio or TV playing, or (4) an inability to hear well enough to carry on a conversation in a quiet room [13,26]. People who identified themselves as deaf were not considered as having hearing loss because their experiences of aging differ from those who acquired hearing loss due to aging.

#### 2.2.3. Neighborhood Disorder

Quality neighborhoods encourage the social engagement of older adults [15]. Neighborhood disorder, one of the moderating variables, consists of three items on a four-point scale (0 = none; 1 = a little; 2 = some, and 3 = a lot). The interviewers rated three contexts (i.e., litter, broken glass, or trash, on sidewalks and streets; graffiti on buildings and walls; and vacant or deserted houses or storefronts) by answering the following question: ‘When standing in front of the respondent’s home/building, and looking around in every direction, how much of the following did you see?’. The first wave of NHATS had a question on the presence of houses with foreclosure signs to measure neighborhood disorder, but the second and third waves did not include that question. Thus, only three aspects of neighborhood disorder were included in this study. The sum of the scores ranged from 0 to 9, with higher scores indicating more observed disorder in the neighborhood. Those whose replies were inapplicable, missing, or non-observable were all considered missing values in the analyses.

#### 2.2.4. Perceived Social Cohesion

Another moderating variable, perceived social cohesion, was measured with three items on a three-point scale (0 = do not agree; 1 = agree a little; and 2 = agree a lot). Individuals were asked whether they agreed with the following statements: ‘People in this community know each other very well’, ‘People in this community are willing to help each other’, and ‘People in this community can be trusted’ [27,28]. The sum of the scores ranged from 0 to 6, with higher scores indicating that individuals perceived greater social cohesion about their communities.

#### 2.2.5. Covariates

Referring to previous population-based and real-world studies [29,30], demographic variables and health variables were included as the covariates. Age, sex, educational level, living alone, race/ethnicity, and annual total income belonged to demographic variables, and self-rated health and the number of chronic diseases consisted of health-related variables. We used age, sex, educational level, race/ethnicity, and total income from Round 1 for the analyses. However, variables, such as living alone, self-rated health, and the number of chronic diseases were treated as time-varying variables in the analyses, using the values of all three waves.

Age was categorized into five groups and included as a continuous variable (1 = 65–69; 2 = 70–74; 3 = 75–79; 4 = 80–84; and 5 = 85–89). Sex was coded as a dichotomous variable, recoding male as a reference group (0 = male; 1 = female). Educational level was coded as a continuous variable (1 = less than high school; 2 = high school graduate; and 3 = some college or more). Race and ethnicity were combined as one variable and categorized into three types: non-Hispanic White (reference group), non-Hispanic Black, and Hispanic. As the sample size of non-Hispanic other groups was limited, we treated them as missing. The annual total income was measured in dollars and log-transformed using the response to the question: “Now think about that total income from Social Security or Railroad Retirement, Supplemental Security Income, the Veteran’s Administration, a pension plan, any retirement accounts, mutual funds or stocks, bonds, bank accounts, CDs, business, farm or real estate, jobs and from any other sources. How much was your and your spouse/partner’s total income before taxes for last year (this is, for the 12 months ending in December)?” Living alone measured the living arrangement of the individuals, determining whether they lived alone or with others (1 = living alone; 0 = living with someone).

Regarding the health-related variables, self-rated health was measured with a single item asking: ‘Would you say that in general, your health is excellent, very good, good, fair, or poor?’ The answer was reversely coded, with a higher score indicating better perceived health (0 = poor; 1 = fair; 2 = good; 3 = very good; and 4 = excellent). The number of chronic diseases was measured as the summation of chronic diseases including heart attack, heart disease, high blood pressure, arthritis, osteoporosis, diabetes, lung disease, stroke, and cancer, ranging from 0 to 9.

### 2.3. Data Analyses

This study used random intercept models to examine whether hearing loss is associated with social isolation over three waves and whether such a relationship is moderated by neighborhood disorder and perceived social cohesion. First, a random intercept model was run using hearing loss, neighborhood disorder, and perceived social cohesion (Model 1). The random option in the random intercept model was used to adjust for within-subject effects across three rounds. The second model included covariates in addition to the first model (Model 2). Then, the final model added two interaction terms: between hearing loss and neighborhood disorder, and between hearing loss and perceived social cohesion (Model 3).

NHATS is a multistage sample design and provides first-stage stratum and cluster variables using the Taylor series linearization [31,32,33,34]. The Round 1–3 analytic weights were tied to the 2010 frame [21], so the baseline’s stratum and cluster variables were used with round-varying analytic weights to define the survey design and reproduce nationally representative estimates of those who were alive and eligible for the survey during the prior round fieldwork period. All statistical analyses were conducted using Stata 13.0 [35].

## 3. Results

Table 1 shows the characteristics of unweighted analytic samples at baseline (Round 1). At baseline, 12.61% (*n* = 498) of community-dwelling older adults reported hearing loss. Among those with hearing loss, about 57% were male and the majority were aged 75 years and above, while 60% of those without hearing loss were female and about half were aged below 75 years. On average, older adults with hearing loss reported their health as being better and perceived their neighborhood as slightly more cohesive than those without hearing loss. Compared to older adults without hearing loss, they lived in a community with less physical neighborhood disorder and were less isolated.

According to the null model on social isolation, the nesting structure was found in the data, with the individual as level 2 and time as level 1 (Table 2). In Model 1 without covariates, hearing loss was not statistically significantly associated with social isolation. However, when we adjusted the model with covariates in Model 2, older adults with hearing loss were significantly less socially isolated (B = −0.01; 95% CI = [−0.18, −0.03]). These results are the opposite of the first research hypothesis, which assumed older adults with hearing loss to be more socially isolated than those without hearing loss.

In Model 3, we investigated the interaction between hearing loss and neighborhood disorder, as well as hearing loss and perceived social cohesion, to determine whether environmental factors modified the association between hearing loss and social isolation. There was statistically significant evidence of an interaction between hearing loss and perceived social cohesion (B = −0.05; 95% CI = [−0.08, −0.01]). However, there was no statistically significant interaction between hearing loss and neighborhood disorder.

**Figure 1:**
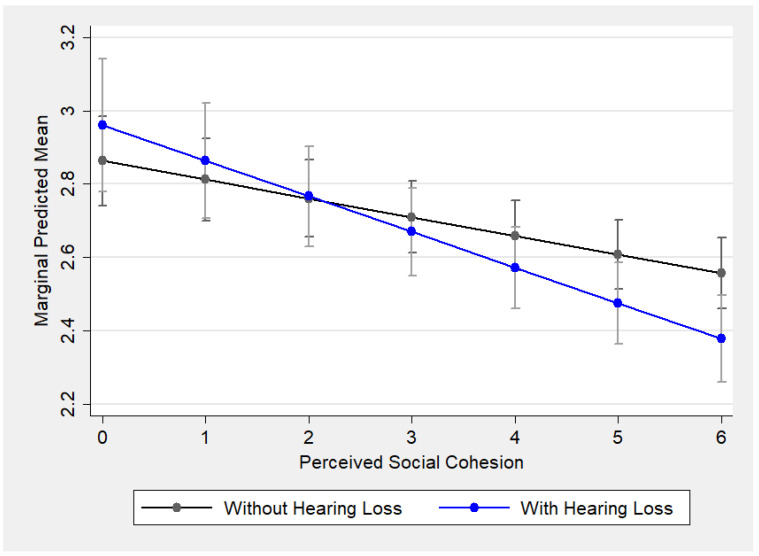
Predictive marginal means of social isolation by hearing loss across the levels of perceived social cohesion in the random intercept model (Model 3).

Figure 1 shows the predicted marginal means of social isolation for older adults with (blue line) and without (black line) hearing loss, respectively, while holding the rest of the variables in the model at their mean values. The *y*-axis represents social isolation scores. Both lines showed a similar downward trend, indicating that higher perceived social cohesion is associated with lower social isolation for both groups. Notably, the two lines crossed, suggesting that the effect of hearing loss on social isolation depends on the value of perceived social cohesion. When the perceived social cohesion was lower (below two points), older adults with hearing loss experienced more social isolation than those without hearing loss. On the contrary, when perceived social cohesion was higher (above two points), older adults with hearing loss were less socially isolated than those without hearing loss.

**Figure 2:**
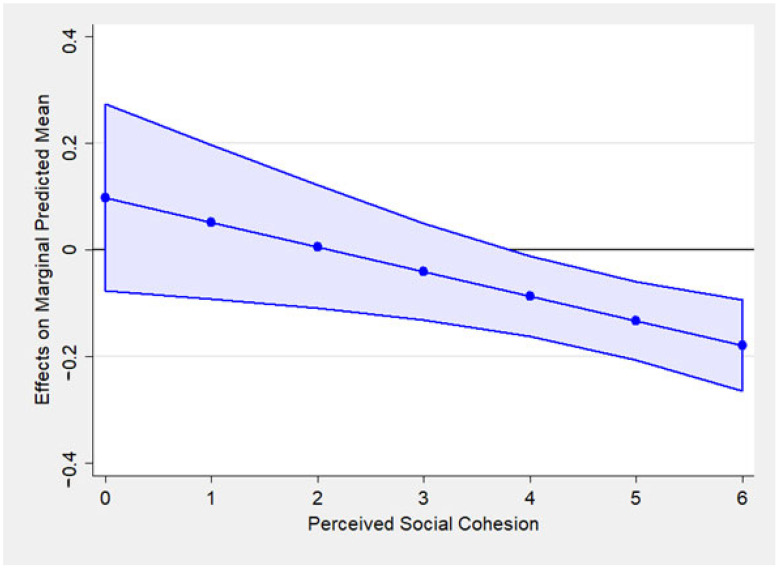
The moderating role of perceived social cohesion on the effect of hearing loss on the marginal predicted mean of social isolation in the random intercept model (Model 3).

Figure 2 shows the marginal effect of hearing loss on social isolation across varying levels of perceived social cohesion. Compared to Figure 1, the *y*-axis represents the effect size, not the social isolation scores, indicating how much hearing loss influenced social isolation at each level of perceived social cohesion. The downward slope indicates that the effect of hearing loss on social isolation decreases as the perceived social cohesion increases. This suggests that higher perceived social cohesion weakens the link between hearing loss and social cohesion. The shaded region shows the confidence interval. If the confidence interval crosses zero on the *y*-axis, the effect is not statistically significant. At lower levels of perceived social cohesion (below four points), the effect of hearing loss appears to be stronger with higher y-values, while it is statistically not significant. On the other hand, at higher levels of perceived social cohesion (above four points), those with hearing loss were less socially isolated than those without age-hearing loss with statistical significance. This pattern suggests that perceived social cohesion has a moderating effect, potentially buffering the negative impact of hearing loss on social isolation at higher levels. Overall, the findings regarding the interaction terms suggest that perceived social cohesion is a protective factor against social isolation among older adults with hearing loss.

## 4. Discussion

This study examined whether hearing loss is associated with social isolation and whether neighborhood disorder and perceived social cohesion moderate the association between hearing loss and social isolation in a nationally representative sample of community-dwelling older adults aged 65 and older. It was found that older adults with hearing loss were less socially isolated than those without hearing loss. This finding is different from what has been found about hearing loss and social isolation. Studies have demonstrated that hearing loss limits the ability to communicate with others and is known as a risk factor for poor psychosocial functioning among older adults [9,36,37,38]. However, such negative effects of hearing loss on social isolation or loneliness can be ameliorated by using hearing aids [39]. The sample in this study all used a hearing aid or other assistive hearing device in the last month before they were interviewed, and this may have affected the difference in the results on the association between hearing loss and social isolation among older adults.

The finding that older adults with hearing loss were less socially isolated than those without may be due to other confounding factors. In this regard, this study found that the effect of hearing loss on social isolation significantly depends on the value of perceived social cohesion. The marginal effects plot (Figure 2) suggested that older adults with hearing loss were less socially isolated than those without hearing loss at higher levels of perceived social cohesion. On the contrary, those with hearing loss were more socially isolated when perceived social cohesion was lower, which is consistent with the previous literature on the relationship between hearing loss and social isolation. Weinstein and Ventry [38] measured the objective and subjective aspects of social isolation. Subjective isolation refers to reactions to barriers to social networks, feelings of loneliness, reduced interest in social engagement, and a desire to stay away from others [38]. While this study’s social isolation focuses on objective aspects, social cohesion measures how close and cohesive older adults subjectively perceive people in their community to be. In other words, scoring high on social cohesion implies that older adults are likely to feel less isolated in a social context. Weinstein and Ventry [38] found that hearing status was more highly correlated with subjective than objective isolation [39]. Thus, high values for social cohesion may attenuate the effect of hearing loss on objective social isolation, explaining the result of the moderating effect of social cohesion.

The current study has several strengths, including the usage of a nationally representative sample of community-dwelling older adults, consideration of a longitudinal approach, and inclusion of environmental factors. However, this study also had some limitations. First, due to the constraints of secondary data availability, the measurement of hearing loss was dichotomous. Studies on hearing loss in older adults have considered the variability in hearing loss by auditory level, physical or social difficulties, or the usage of a hearing aid [8,40,41]; however, this study separated community-dwelling older adults into two groups solely based on the use of a hearing aid or other hearing device in the last month. This does not capture the length for which the hearing aid was used. The first-time users’ experience may be different from those who have already adjusted to the device [42]. In addition, among the older adults who reported that they had not used a hearing aid or device in the last month, there may be some people who had not used a hearing aid recently or who were not financially able to purchase the device but who had experienced hearing loss [43]. A recent study found that higher-income older adults are more likely to use hearing aids, highlighting the role of healthcare access and economic factors [29]. Given the high cost, lower-income older adults may delay obtaining one. There may also be cultural barriers to hearing aid access. Stigma may prevent older adults with hearing loss from using hearing aids. Internalized and externalized stigma can make them perceive hearing aids as a sign of impairment [16,44]. Future research should explore how stigma influences the link between hearing loss and social isolation.

Additionally, due to the limitations of using secondary data, this study used neighborhood disorder and social cohesion to investigate the effect of environmental factors on social isolation based on the ecological model of aging. However, these variables were measured from self-reported observations and perceptions of neighborhood characteristics rather than objective neighborhood characteristics. To examine the neighborhood effect on the individual outcome, future studies need to consider combining the current data with geographic or any other neighborhood-level information. Social isolation among older adults with hearing loss is a complex issue. Hearing loss varies by race/ethnicity, sex, socioeconomic status, and insurance coverage [29], leading to unique experiences of social isolation. Future research should explore how the relationship between hearing loss, social isolation, and environmental factors differs across diverse socio-demographic backgrounds. While more recent NHATS data are available, this study focused on examining the relationship between hearing loss, social isolation, and neighborhood characteristics using earlier waves. Recent research has reinforced the association between hearing loss and social isolation [13] and has also confirmed that social cohesion moderates the relationship between hearing loss and cognition [45]. Thus, this study serves as a baseline study for understanding the relationship between hearing loss, social isolation, and environmental factors.

## 5. Conclusions

This study is an important addition to the literature regarding hearing loss. The findings illustrate the longitudinal association between hearing loss and social isolation and the significant differences in the effect of hearing loss on social isolation according to the level of perceived social cohesion. While hearing function deteriorates as individuals age, hearing aids or other hearing devices are rarely covered by third parties other than some state Medicaid plans or rehabilitation services for veterans [46]. Additionally, the Medicare Annual Wellness Visit offers a key chance to address hearing concerns, but the wording of its assessment may discourage disclosure. The binary question regarding deafness or serious trouble hearing can lead older adults to withhold concerns. The research highlights the need for educational programs to improve clinician–patient communication [47]. To allow for its broader application, addressing modifiable neighborhood factors may be one of the most effective strategies to promote social inclusion for older adults with hearing loss. For instance, policies should prioritize the development of age-friendly neighborhoods by ensuring accessible gathering spaces are available, increasing the availability of real-time audio information in public transportation, and fostering community programs that facilitate social interactions for individuals with hearing loss [48,49,50]. The literature suggests that hearing loss not only restricts older adults from social participation and activities but also increases their reliance on formal support or community services [51,52]. This study only focused on the general impression of individuals’ surrounding neighborhoods and their perceptions of their community. Thus, future studies should continue to investigate the effect of environmental factors on individual outcomes and should examine in detail which structures and services of their neighborhoods are supportive to older adults with hearing loss.

## Figures and Tables

**Table 1:** Sample characteristics at Round 1.

	Total Analytic Sample		With Hearing Loss		Without Hearing Loss	
Variables	M (SD)/%	N *	M (SD)/%	N *	M (SD)/%	N *
Age category, %						
65–69 years old	21.97	868	7.43	37	24.07	831
70–74 years old	23.67	935	15.46	77	24.86	858
75–79 years old	21.24	839	21.29	106	21.23	733
80–84 years old	19.34	764	25.1	125	18.51	639
85–89 years old	9.65	381	20.68	103	8.05	278
90+ years old	4.13	163	10.04	50	3.27	113
Female, %	58.18	2298	42.77	213	60.4	2085
Education level, %						
Less than HS	22.76	899	19.88	99	23.17	800
HS graduate	49.67	1962	52.61	262	49.25	1700
Some college +	27.57	1089	27.51	137	27.58	952
Race/ethnicity, %						
Non-Hispanic White	73.9	2919	88.55	441	71.78	2478
Non-Hispanic Black	20.94	827	8.03	40	22.8	787
Hispanic	5.16	204	3.41	17	5.42	187
Living alone, %	32.13	1269	32.93	164	32.01	1105
Medicaid receipt, %	11.96	468	8.08	40	12.53	428
Annual total income	$29,901.34 (2.84)	3950	$34,452 (2.24)	498	$29,313 (2.92)	3452
Self-rated health	2.32 (1.08)	3949	2.38 (0.99)	498	2.31 (1.09)	3451
Chronic diseases	2.47 (1.51)	3922	2.53 (1.49)	495	2.46 (1.52)	3427
Neighborhood disorder	0.28 (0.91)	3948	0.14 (0.52)	498	0.3 (0.95)	3450
Perceived social cohesion	4.27 (1.64)	3773	4.54 (1.56)	476	4.23 (1.65)	3297
Social isolation	2.41 (1.19)	3934	2.26 (1.15)	496	2.43 (1.2)	3438

Note. * N refers to the number of individuals who responded to the variable. HS = High school.

**Table 2:** Random intercept multilevel models on social isolation over three waves ^a^.

Outcome: Social Isolation	Unconditional Null Model	Main Effect (Model 1)	With Covariates (Model 2)	With Interactions Terms (Model 3)
Intercept	2.3 ***	2.53 ***	4.34 ***	4.32 ***
Time	0.01	0.01	−0.01	−0.004
Hearing loss		−0.05	−0.1 **	0.1
Neighborhood disorder		0.06 ***	0.05 **	0.05 **
Social cohesion		−0.06 ***	−0.06 ***	−0.05 ***
Age			0.08 **	0.09 **
Sex (ref: male)			−0.21 ***	−0.21 ***
Education level			−0.11 ***	−0.12 ***
Race/ethnicity (ref: White)				
Black			0.14 ***	0.14 ***
Hispanic			0.24 **	0.24 **
Medicaid receipt			0.13 ***	0.13 ***
Annual total income			−0.15 ***	−0.15 ***
Self-rated health			−0.08 ***	−0.08 ***
Chronic diseases			0.002	0.002
Living alone			0.49 ***	0.49 ***
ND * ARHL				−0.019
PSC * ARHL				−0.05 *
Variance components				
Level 1 variance	0.34	0.34	0.34	0.34
Level 2 variance	1.05	0.99	0.73	0.73
ICC	0.75	0.74	0.68	0.68

Note. ^a^ All models show unstandardized coefficients. ND = neighborhood disorder; PSC = perceived social cohesion; ref = reference group; ICC = intraclass correlation. * *p* < 0.05, ** *p* < 0.01, *** *p* < 0.001.

## Data Availability

The original data presented in the study are openly available at www.nhats.org (accessed on 1 July 2021).
